# IV and oral fosfomycin pharmacokinetics in neonates with suspected clinical sepsis

**DOI:** 10.1093/jac/dkab083

**Published:** 2021-04-14

**Authors:** Zoe Kane, Silke Gastine, Christina Obiero, Phoebe Williams, Sheila Murunga, Johnstone Thitiri, Sally Ellis, Erika Correia, Borna Nyaoke, Karin Kipper, John van den Anker, Mike Sharland, James A Berkley, Joseph F Standing

**Affiliations:** 1 Infection, Immunity and Inflammation, Great Ormond Street Institute of Child Health, University College London, London, UK; 2 Quotient Sciences, Mere Way, Ruddington, Nottingham, UK; 3 KEMRI-Wellcome Trust Research Programme, Kilifi, Kenya; 4 Centre for Tropical Medicine & Global Health, Nuffield Department of Medicine, University of Oxford, Oxford, UK; 5 GARDP—Global Antibiotic Research & Development Partnership, Genève, Switzerland; 6 DNDi—Drugs for Neglected Diseases initiative, Nairobi, Kenya; 7 Institute of Chemistry, University of Tartu, Tartu, Estonia; 8 Department of Paediatric Pharmacology and Pharmacometrics, University Children’s Hospital Basel, University of Basel, Basel, Switzerland; 9 Division of Clinical Pharmacology, Children’s National Hospital, Washington, DC, USA; 10 Paediatric Infectious Diseases Research Group, Institute for Infection and Immunity, St George’s, University of London, London, UK; 11 The Childhood Acute Illness & Nutrition (CHAIN) Network, Nairobi, Kenya; 12 Pharmacy Department, Great Ormond Street Hospital for Children, NHS Foundation Trust, London, UK

## Abstract

**Background:**

Fosfomycin has the potential to be re-purposed as part of a combination therapy to treat neonatal sepsis where resistance to current standard of care (SOC) is common. Limited data exist on neonatal fosfomycin pharmacokinetics and estimates of bioavailability and CSF/plasma ratio in this vulnerable population are lacking.

**Objectives:**

To generate data informing the appropriate dosing of IV and oral fosfomycin in neonates using a population pharmacokinetic analysis of plasma and CSF data.

**Methods:**

The NeoFosfo study (NCT03453177) was a randomized trial that examined the safety and pharmacokinetics of fosfomycin comparing SOC versus SOC plus fosfomycin. Sixty-one neonates received fosfomycin (100 mg/kg IV q12h for 48 h) and then they converted to oral therapy at the same dose. Two plasma pharmacokinetic samples were taken following the first IV and oral doses, sample times were randomized to cover the whole pharmacokinetic profile and opportunistic CSF pharmacokinetic samples were collected. A population pharmacokinetic model was developed in NONMEM and simulations were performed.

**Results:**

In total, 238 plasma and 15 CSF concentrations were collected. A two-compartment disposition model, with an additional CSF compartment and first-order absorption, best described the data. Bioavailability was estimated as 0.48 (95% CI = 0.347–0.775) and the CSF/plasma ratio as 0.32 (95% CI = 0.272–0.409). Allometric weight and postmenstrual age (PMA) scaling was applied; additional covariates included postnatal age (PNA) on clearance and CSF protein on CSF/plasma ratio.

**Conclusions:**

Through this analysis a population pharmacokinetic model has been developed that can be used alongside currently available pharmacodynamic targets to select a neonatal fosfomycin dose based on an infant’s PMA, PNA and weight.

## Introduction

Antimicrobial resistance is a global health priority and neonates are a particularly vulnerable population. In 2013, infection accounted for one-quarter of all neonatal deaths globally;^1^ in Asia and Africa, 50%–88% of clinical isolates are reported to be resistant to the first-line antibiotics ampicillin and gentamicin.^2^^,^^3^ Fosfomycin, an affordable and effective antibiotic, has emerged as one potential solution.^4^^,^^5^

It has recently been shown that community-acquired Gram-negative bacteraemia isolates are significantly more likely (96% versus 59%; *P *<* *0.0001) to be susceptible to fosfomycin than empirical ampicillin/gentamicin therapy.^6^ Therefore, used in combination with another appropriate antibiotic, fosfomycin may present an alternative treatment strategy for empirical management of hospital- or community-acquired MDR Gram-negative sepsis in neonates. However, optimal fosfomycin dosing in neonates is currently uncertain, including whether oral therapy is feasible.^5^ The limited neonatal pharmacokinetic (PK) studies that have been published are mostly 30 to 40 years old,^7–10^ small studies (∼10 subjects) and only evaluated IV fosfomycin PK. Oral fosfomycin PK and consequently bioavailability (F) has never previously been reported for a neonatal population. Predicting neonatal oral PK is challenging since in adults F depends on the fosfomycin salt form and physiological gastrointestinal conditions.^11^^,^^12^ Finally, neonates suffering from sepsis may also have bacterial meningitis, so evaluating the extent of fosfomycin CNS penetration is highly desirable.

In adults, 85%–95% of a fosfomycin dose is excreted unchanged in the urine^13^ with clearance similar to glomerular filtration rate (GFR), the volume of distribution is 0.42 L/kg, the half-life is 2.4–2.8 h and the bioavailability is 0.53 for a 3 g dose of the tromethamine salt, a regimen that sees plasma levels sustained >1 mg/L for 48 h due to absorption rate limited elimination;^14^ others have also confirmed flip-flop kinetics.^15^ Because of fosfomycin’s low molecular mass, and despite its hydrophilicity, it enters the CNS regardless of meningeal inflammation.^16^^,^^17^

Here we report on the model-based estimation of fosfomycin IV and oral plasma and CSF PK from a trial in neonates with suspected clinical sepsis.

## Methods

### Study and drug details

The NeoFosfo study (ClinicalTrials.gov: NCT03453177) ran between March 2018 and March 2019 at Kilifi County Hospital in Kenya. Neonates aged 0 to 28 days old, weighing >1500 g and born at >34 weeks of gestation (using Ballard Maturational Assessment) with at least one sign of clinical sepsis and eligible to receive IV antibiotics (according to national guidelines) were included. Neonates were randomized to standard of care (SOC) consisting of ampicillin and gentamicin or SOC plus 100 mg/kg fosfomycin twice daily (q12h). Patients in the fosfomycin arm received a minimum of four IV fosfomycin doses (Fomicyt 40 mg/mL solution, Infectopharm, Germany) over 48 h at 12 h intervals. Once oral fluids were tolerated, IV fosfomycin was changed to oral fosfomycin (Fosfocina 250 mg/5 mL suspension, ERN Laboratories, Spain) therapy at 100 mg/kg q12h. The IV dose was given as a slow push, while oral fosfomycin was given via oral syringe, spoon or nasogastric tube. Once reconstituted, fosfomycin was stored below 25°C. The dose of 100 mg q12h was selected for use in the NeoFosfo study based on the current age and weight-based neonatal dosing recommendations in the Fomicyt summary of product characteristics (‘SPC’) and the findings of Traunmuller *et al*.^18^

### PK sampling

Simulation-based sample size calculations were performed.^19^^,^^20^ A cross-over study design with each subject providing two IV and two oral plasma samples was predicted to estimate clearance, central volume and bioavailability with a power of >85% to have 95% CIs within a 20% precision level if 45 patients were included. Due to uncertainty regarding the shape of the PK profile, patients were randomized to a single early (0.08, 0.5 or 1 h post-dose) and single late (2, 4 or 8 h post-dose) PK sample following the first IV and oral doses. Patients who remained hospitalized underwent a final safety blood sample following the last oral dose at day 7 and, if there was sufficient blood volume drawn, this was also assayed for PK. In addition, if a lumbar puncture was undertaken for clinical investigation during treatment, fosfomycin concentration was also measured in the CSF.

### Fosfomycin bioanalysis

Plasma and CSF samples were centrifuged at 3000 rpm for 5 min then separated and frozen (at −80°C) within 30 min of collection. Frozen samples were shipped to Analytical Services International Ltd, St Georges University of London, UK. Analysis of fosfomycin concentration in plasma and CSF samples was assessed via LC-MS/MS assay. The lower limit of quantification for plasma was 5 mg/L and for CSF was 1 mg/L. The method was fully validated according to EMA guidlines.^21^ Assay methodology and fosfomycin stability data can be found in the Supplementary data available at *JAC* Online.

### PK model development

Model-based estimation of PK parameters was undertaken using the first-order conditional estimation method with interaction (‘FOCEI’) in NONMEM (Version 7.4; ICON Development Solutions, Ellicott City, MD, USA).

One- and two-compartment structural models were compared. Inter-individual variability (IIV) was assumed to follow a log-normal distribution for clearance, volume and absorption rate constants, and a logit distribution for bioavailability. Estimation of IIV was evaluated for all parameters. An additive, a proportional and a combined error model were tested. In line with the χ^2^ distribution, a drop in the log likelihood ratio of >6.64 per degree of freedom was needed to be significant at a level of *P *<* *0.01 and >3.84 at a level of *P *<* *0.05.

Allometric (weight) scaling was included using a fixed exponent of 0.75 on clearance terms and linear scaling on volume terms. A standard weight of 70 kg was used to enable comparison of parameter estimates with other studies. A previously published neonatal renal maturation function^22^ was also added to clearance. Due to the narrow postmenstrual age (PMA) range of babies included in this study the Hill coefficient and time to 50% maturation were fixed as with previous similar neonatal studies.^23^^,^^24^

$$CL_{i}=CL_{\mathrm{std}}\times {\left(\frac{WT_{i}}{70}\right)}^{0.75}$$

$$V_{i}=V_{\mathrm{std}}\times \left(\frac{WT_{i}}{70}\right)$$

$$\mathrm{maturation}=\frac{{PMA_{i}}^{3.4}}{47.7^{3.4}+{PMA_{i}}^{3.4}}$$

While the Rhodin maturation function^22^ accounts for development of renal maturation in early life, there may also be a further effect on clearance maturation after birth regardless of gestational age (GA) that occurs over the first few days/weeks of life.^25^ This covariate may be best related to postnatal age (PNA) as has been observed by others.^23^^,^^26^ Therefore Equation 4 was also evaluated (θ_M_; fraction of clearance on the first day of life, set to day = 0; and θ_N_, postnatal maturation rate constant).

$${\mathrm{PNA}}_{\mathrm{function}}=\theta_{M}+\left(1-\theta_{M}\right)\times \left(1-e^{-PNA_{i}\times \theta_{N}}\right)$$

The ability of serum creatinine concentration (SCR) to explain and reduce IIV on clearance was tested according to Equation 6, where the measured SCR was standardized using typical serum concentration (TSCR) for age calculated based on the function published by Ceriotti *et al*.^27^ (Equation 5). The SCR levels utilized by Ceriotti *et al*.^27^ in defining Equation 5 were quantified using an enzymatic method, while a Jaffe method was used in this study.

$$\mathrm{TSCR}\left(\mu \mathrm{mol}\right)=-2.37330-12.91367\times \ln({\mathrm{PNA}}_{\mathrm{years}})+23.93581\times {\left({\mathrm{PNA}}_{\mathrm{years}}\right)}^{0.5}$$

$${\mathrm{SCR}}_{\mathrm{function}}={\frac{SCR_{i}}{\mathrm{TSCR}}}^{\theta_{\mathrm{SCr}}}$$

#### CSF modelling

Having defined the plasma population PK model including covariate effects, an additional peripheral compartment was added to model the available CSF data.^28^^,^^29^ This introduced three additional parameters: inter-compartmental clearance between the central and CSF compartments (Q2), volume of the CSF compartment (*V*4) and the CSF/plasma ratio (UPTK).

The initial modelling strategy aimed to estimate Q2 and UPTK, while the volume of the CSF compartment was fixed to 0.15 L/70 kg with linear weight scaling.^30^ However, due to model instability, published adult CSF data^16^ were used to define and subsequently fix Q2 in the NeoFosfo model [see Figure S1 (available as Supplementary data at *JAC* Online) and the Discussion section].

CSF protein was tested as a covariate on UPTK according to Equations 7 to 9, where PR_i_ is the individual’s measured CSF protein level and 0.94 the population’s median CSF protein level.

$$\mathrm{UPTK}1=\ln\left(\frac{\theta_{\mathrm{UPTK}}}{1-\theta_{\mathrm{UPTK}}}\right)$$

$$\mathrm{UPTK}2=\mathrm{UPTK}1\times \left(\left(1+\theta_{PR}\right)\times \left(PR_{i}-0.94\right)\right)$$

$$\mathrm{UPT}K_{i}=\frac{1}{1+e^{-\mathrm{UPTK}2}}$$

#### Model evaluation

Decisions during model development were made based on the likelihood ratio test, goodness of fit (GOF) plots^31^ and visual predictive checks (VPCs) using *n *=* *1000 simulations. A non-parametric bootstrap (*n *=* *1000) was performed on the final model to test parameter robustness and derive parameter uncertainty. Perl-speaks-NONMEM (PsN)^32^ was used for the bootstrap analysis and to produce the VPCs, which were visualized using Xpose4.^33^

### PK simulations

Using the final model parameter estimates, the half-life for each phase of the PK profile was calculated using methods reported by Upton.^34^

A hypothetical neonatal population of 10 000 subjects was created using observed demographics from the present study combined with data from an international multicentre neonatal observational study (NeoObs study; NCT03721302). The hypothetical population along with the final model were used to simulate fosfomycin plasma concentrations at steady-state for different dosing regimens. The simulations were all executed in R using the linpk package.^35^

Target attainment (TA) plots were generated considering two potentially relevant pharmacodynamic targets: AUC/MIC ratio^36^ and *T*_>MIC_.^18^ Target values for *T*_>MIC_ were defined (60%, 80% and 100%), but, for the AUC/MIC ratio, previously published *in vitro* target values for *Escherichia coli* were considered [stasis (19.3), 1 log kill (87.5)^37^ and resistance suppression (3136)].^38^ Due to uncertainty regarding the specific target value required for predicting the efficacy of fosfomycin in bacteraemia, here we use an approach previously suggested^39^ and present TA rather than ‘PTA’ against ascending MIC values.

## Results

### Patients and demographics

Sixty-one babies were recruited into the SOC plus fosfomycin arm of the study, with fosfomycin PK sampling performed for 60 babies. Demographics are given in Table 1.

**Table 1.** Key baseline covariates of all patients receiving fosfomycin in the NeoFosfo study compared with the NeoObs study

	NeoFosfo study (NCT03453177)		NeoObs study (NCT03721302)	
	median (range)	mean (IQR)	median (range)	mean (IQR)
GA (weeks)	40.0 (34.4–44.0)	39.8 (38.4–40.8)	37 (23–44)	35.4 (31–39)
PNA (days)	1 (0–23)	2.7 (0–3)	5 (0–59)	10.4 (2–15)
Weight (g)	2805 (1560–5670)	2872 (2500–3234)	2500 (400–5170)	2353 (1400–3197)
PMA (weeks)	40.1 (34.5–46.2)	40.3 (38.7–41.3)	38.1 (23.1–51.9)	36.8 (32.4–40.3)
CSF protein (g/L)	0.94 (0.02–2.51)	1.07 (0.78–1.16)	0.92 (0.004–9.14)	1.24 (0.64–1.39)

### Observed PK data

In total, 238 fosfomycin plasma samples were collected. Two babies died during the IV fosfomycin phase of the study resulting in complete IV and oral PK data being collected for 58 babies. Plasma protein binding of fosfomycin is negligible^40^^,^^41^ and therefore calculation of free concentrations was not necessary.

Fifteen CSF samples from 15 subjects were collected. Five of the CSF samples were collected during IV treatment and 10 of the CSF samples were collected during oral treatment. PR_i_ values were available for 12 subjects.

No samples (plasma or CSF) were below the limit of quantification.

**Figure 1.**
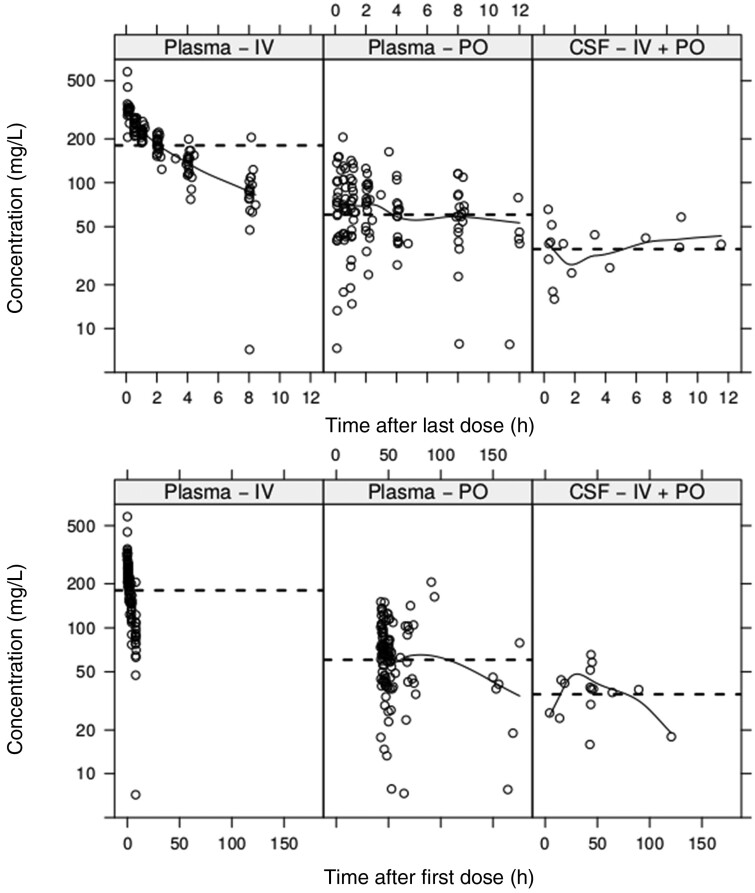
Observed CSF and plasma concentration versus time data for all subjects. The dashed lines represent mean concentrations, which are 37.6 mg/L in CSF and 70.1 and 201.7 mg/L in plasma following oral and IV dosing, respectively; the solid lines represent the loess smooth curves. PO, oral.

Plasma and CSF concentration data are presented in Figure 1. Individual subject PK plots (plasma and CSF) are shown in Figures S2 and S3.

### PK model development

A two-compartment IV structural model was superior to a one-compartment model (−ΔOFV 31, plus two degrees of freedom, *P *<* *0.01; where OFV stands for objective function value). When modelling the IV data in isolation it was only possible to estimate IIV on CL; however, increasing the dataset to include the oral plasma data enabled estimation of IIV on CL, volume of the central compartment (*V*c) and F (−ΔOFV 22, no change in degrees of freedom, *P *<* *0.01).

The correlations between key demographic and biochemical covariates were analysed and correlation coefficients calculated (see Figure S4). The strongest correlation was observed between SCR and PNA (*r*^2^ = 0.52); SCR showed a slight initial increase with PNA followed by a rapid then slower decline. Weight correlated to a lesser extent with the different measures of age (PNA, *r*^2^ = 0.32; PMA, *r*^2^ = 0.33), whilst PMA and SCR appeared essentially independent (*r*^2^ = 0.1).

Inclusion of allometric scaling and the Rhodin maturation function resulted in a drop in OFV of 84 (fixed covariate functions, no additional degrees of freedom) and inclusion of the PNA function described in Equation 4 gave a further OFV decrease of 43 (plus two degrees of freedom, *P *<* *0.01). However, inclusion of SCR instead of PNA, or in conjunction with PNA, did not improve the model compared with just using allometric scaling and the Rhodin function (−ΔOFV by <1, for one degree of freedom). CSF protein was found to be a significant covariate for the CSF/plasma ratio (UPTK) reducing the OFV by 4.6 for one additional degree of freedom (*P *<* *0.05).

**Figure 2.**
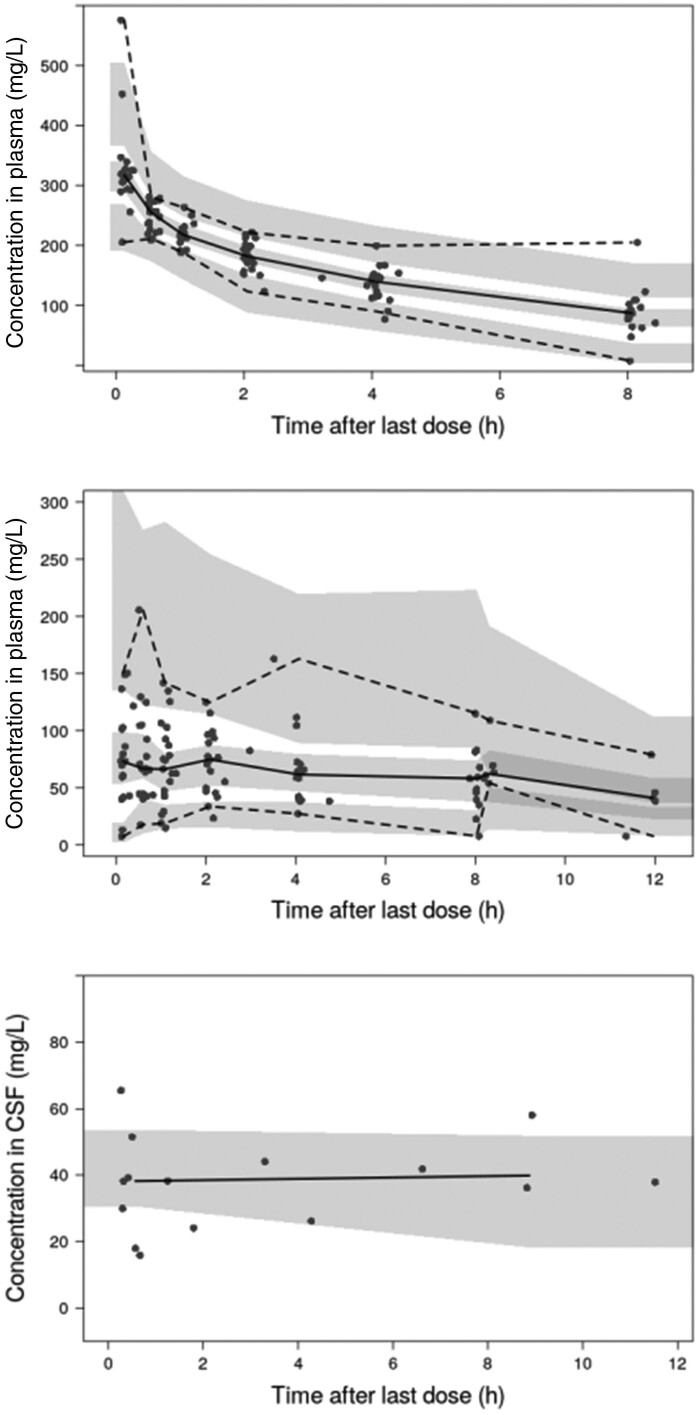
VPCs showing the observed data (black circles) and the 2.5th, 50th and 97.5th percentiles of the observed data (black lines) compared with the 95% CIs of the corresponding simulations (prediction intervals) from the final model (shaded areas). The top panel shows plasma following IV dosing, the middle panel shows plasma following oral dosing and the bottom panel shows CSF. The 2.5th and 97.5th percentiles, and corresponding prediction intervals, are not presented in the bottom panel due to the size of the CSF dataset evaluated.

**Figure 3.**
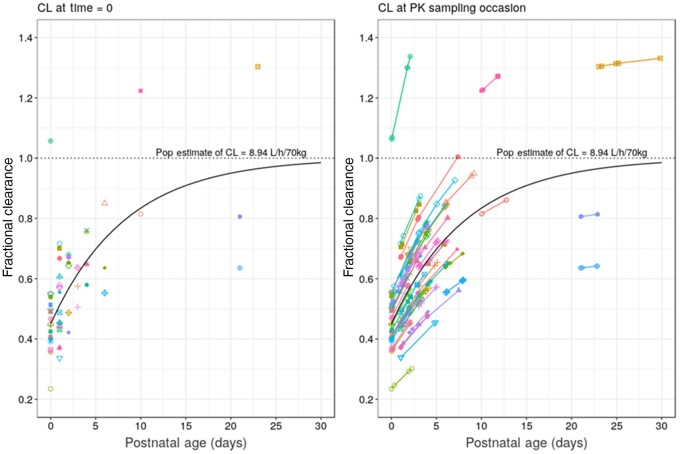
Visualization of the PNA effect on clearance; individual predicted clearances have already been scaled for PMA and weight. Data points are grouped by subject ID (*n *=* *60); the left-hand panel shows individual fractional clearance at time = 0 and the right-hand panel shows individual fractional clearance at all PK sampling timepoints. The solid black line in each panel represents the model estimated PNA maturation function.

An illustration of the final structural model is presented in Figure S5 and model parameters along with their associated relative standard errors (%RSE) and 95% CI are presented in Table 2. The shrinkage in the estimates of IIV on CL, *V*c and F are 4.7%, 17.8% and 47.5%, respectively. VPCs are shown in Figure 2. GOF and individual prediction plots are shown in Figures S6 to S9. The PNA function compared with individual clearance estimates is shown in Figure 3. The final model employed three residual/unexplained error terms. Separate proportional error models were used to describe the IV and oral plasma levels and an additive error model was used for CSF concentrations. The NONMEM code for the final NeoFosfo model is given in the Supplementary data available at *JAC* Online.

**Table 2.** Population PK model parameter estimates; all disposition terms are centred on a fully mature 70 kg individual using allometric scaling with exponents of 1 for volume terms and 0.75 for clearance terms

Parameter	Estimate (%RSE)	IIV %CV (%RSE)	Bootstrap 95% CI	Bootstrap median
CL (L/h/70 kg)	8.94 (14.5)	24.5 (30.5)	7.10 to 13.2	9.13
*V*2 (L/70 kg)	19.1 (8.77)	14.2 (41.8)	11.2 to 21.3	19.0
Q1 (L/h/70 kg)	8.01 (49.6)	–	4.54 to 39.3	8.24
*V*3 (L/70 kg)	7.53 (14.0)	–	5.69 to 14.3	7.61
Q2 (L/h/70 kg)	0.017 (fixed)	–	–	–
*V*4 (L/70 kg)	0.15 (fixed)	–	–	–
θ_UPTK_	0.321 (12.0)	–	0.272 to 0.409	0.32
Ka (/h)	0.0987 (21.7)	–	0.0570 to 0.148	0.0994
F	0.478 (15.0)	0.269 (60.2)	0.347 to 0.775	0.483
θ_M_	0.449 (22.9)	–	0.277 to 0.567	0.420
θ_N_ (/day)	0.117 (29.4)	–	0.0531 to 0.259	0.121
θ_PR_	−0.952 (22.4)	–	−2.88 to −0.615	−1.081
IV plasma proportional error (%)	7.69 (46.4)	–	3.78 to 12.1	8.22
Oral plasma proportional error (%)	18.6 (37.5)	–	7.36 to 24.3	16.6
CSF additive error (mg/L)	10.9 (35.3)	–	5.47 to 14.6	10.2

CL, total plasma clearance; *V*2, central volume; Q1, inter-compartmental clearance between the central and main peripheral compartments; *V*3, volume of the main peripheral compartment; Q2, inter-compartmental clearance between the central and CSF compartments; *V*4, volume of the CSF compartment; θ_UPTK_, CSF/plasma ratio; Ka, absorption rate constant; F, oral bioavailability; θ_M_, population estimate of the fraction of clearance on the first day of life, set to day = 0; θ_N_, postnatal maturation rate constant; θ_PR_, CSF protein coefficient; %RSE, asymptotic standard error; %CV, coefficient of variation; IIV, inter-individual variability.

IIV on F reported directly as OMEGA value due to logit transformation.

The demographics of the hypothetical simulation population are presented in Figure S10. Predicted steady-state PK parameters (AUC, *C*_max_ and *C*_min_) using the final model and hypothetical population are presented in Table S1.

**Figure 4.**
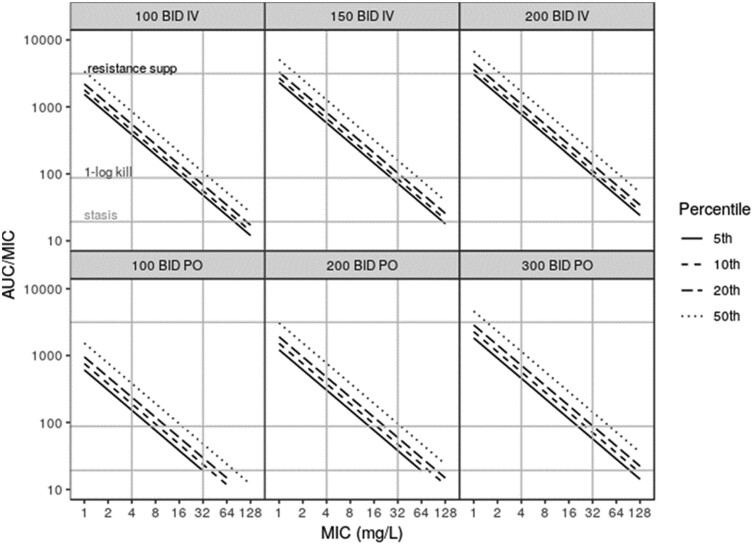
TA plots for various dose schemes using the full simulation population. The top row presents predicted AUC/MIC ratio in plasma following IV dosing, while the bottom row shows results following oral dosing. A comparison of 100, 150 and 200 mg/kg q12h is given for IV and 100, 200 and 300 mg/kg q12h for oral. The continuous black line is the predicted AUC/MIC ratio achieved by 95% of the population (5th percentile), while the typical patient (50th percentile) is shown by the dotted line. AUC/MIC target ratios for stasis (19.3), 1 log reduction (87.5) and resistance suppression (3136) are shown by the grey horizontal reference lines. The grey vertical reference lines highlight MIC values of 4 and 32 mg/L. BID, twice daily; PO, oral.

TA results using the full hypothetical population are presented in Figure 4 (AUC/MIC) and Figure S11 (*T*_>MIC_). For pathogens with an MIC of 32 and 4 mg/L, a q12h 100 mg/kg IV regimen was predicted to achieve a plasma AUC/MIC ratio of 48 and 385, respectively, in 95% of neonates. Following oral dosing of 100 mg/kg q12h and evaluating the same MICs, the predicted plasma AUC/MIC ratios are 19 and 152 in 95% of neonates. Considering *T*_>MIC_, and a q12h 100 mg/kg IV regimen, 95% of neonates are predicted to exceed 32 mg/L 51% of the time. The flatter profile shape that results from slow absorption following oral administration (*T*_max_ = ∼3–4 h) and oral bioavailability means that, at 100 mg/kg q12h, 95% of neonates were predicted to never exceed 32 mg/L; however, 16 mg/L is exceeded 100% of the time.

## Discussion

To the best of our knowledge, we have conducted the first neonatal cross-over bioavailability study of fosfomycin and, with a minimally invasive design, precise population PK estimates have been derived. Our model and simulations should prove useful in determining optimal fosfomycin dosing in neonates.

Our population estimates for CL (8.94 L/h/70 kg) and *V*c (19.11 L/70 kg) are in agreement with PK observed in healthy adults as is the calculated β phase half-life of 2.3 h (after 1 g of IV fosfomycin to healthy adults, *V* = 29.7 ± 5.7 L, CL = 8.7 ± 1.7 L/h, weight = 70.5 ± 11.1 kg, *t*_½_ = 2.4 ± 0.4 h).^14^ The calculated adult equivalent γ phase half-life of 6.1 h, resulting from the additional CSF compartment, is unlikely to be measured clinically. As such, the β phase is considered the clinically relevant plasma half-life. Half-lives for the typical (median) trial neonate (weight = 2805 g, PNA = 1 day, PMA 40 weeks) for each elimination phase were longer than those predicted in a 70 kg adult (neonate: 0.2, 5.2 and 8.7 h; adult: 0.4, 2.3 and 6.1 h).

The Rhodin maturation function^22^ describes the development of renal function in early life and was added to our model as fixed biological prior knowledge, as reported in previous similar neonatal PK studies.^23^^,^^24^ Having adjusting clearance for weight and PMA we still saw a strong relationship between CL and PNA (see Figure 3). The PNA_function_ successfully captured the increases in clearance over the first few days/weeks of life. Nephrogenesis is complete by the 34th–36th week of gestation; however, the functional maturation of the kidney continues through the postnatal period.^42^ The first days/weeks following birth see a rapid increase in renal blood flow as a function of cardiac output, GFR and urine output, all of which are likely to contribute to the significance of PNA as a covariate on fosfomycin clearance.

When considering the TA results, it is important to highlight the difference between the median PNA of the hypothetical population and the NeoFosfo trial population [5 (mean = 10) versus 1 (mean = 3) days, respectively], as, within a given individual, clearance is predicted to increase by 36% over this time frame (based on just this increase in PNA). To illustrate further the implications of PNA on predicted TA, plots analogous to Figure 4 were constructed for neonatal sub-populations categorized by weight and PNA and are presented in Figure S12 for IV dosing and Figure S13 for oral dosing.

SCR was not found to be a significant covariate on fosfomycin clearance. As with previous neonatal antimicrobial PK reports,^23^ we used the function published by Ceriotti *et al*.^27^ to account for expected postnatal changes in SCR. This function accounts for the postnatal decline in SCR due to washout of maternal creatinine and then the subsequent rise with age. Whilst SCR is often used to predict GFR in adults, the relationship between SCR and GFR in the newborn infant is complicated^43^ and measured SCR in neonates <2–3 weeks old is known to be highly variable.^27^ In this study, 75% of babies were <3 days old on admission and we observed a 3-fold range in baseline measured SCR; both of these factors are thought to have contributed to the lack of correlation between fosfomycin clearance and SCR in this study.

Oral bioavailability was estimated to be 0.48 (dose was nominally 100 mg/kg) and, with limited first-pass extraction, this is likely indicative of the fraction absorbed. Fosfomycin is a low molecular weight (138 g/mol), highly polar (ACD/logP = −2.98) phosphonic acid derivative, thereby demonstrating pH-dependent solubility and ionization in the gastrointestinal tract. Fosfomycin absorption is likely to be permeability limited, which is consistent with the class III Biopharmaceutics Drug Disposition Classification assigned by Benet *et al*.^44^*In vitro* intestinal permeability studies suggest fosfomycin is absorbed via both the paracellular and transcellular routes with uptake mediated in part by the Na+-dependent phosphate transport system.^45^^,^^46^ However, the fact that fosfomycin F is increased 3-fold by switching from the calcium to tromethamine salt form does indicate that solubility and/or stability limitations cannot be disregarded.^12^^,^^15^^,^^47^ The calcium salt form was administered in this study (Fosfocina 250 mg/5 mL suspension), which, at the much lower dose of 7.5 mg/kg, is reported to be only 37% bioavailable in adults.^48^ Interestingly, the rate of absorption also seems to be faster than reported in adults. Wenzler *et al*.^14^ reported a Ka of 0.0175/h following administration of 3 g (∼43 mg/kg) of the tromethamine salt in adults, while here we report a population estimate of 0.0987/h, >5-fold faster, which combined with a lower relative clearance due to renal immaturity means flip flop kinetics are not as prominent a feature of fosfomycin PK in the NeoFosfo population as in adults. The elevated rate and extent of absorption seen in this study compared with that reported in adults is attributed to increased permeability of the immature intestinal barrier in neonates <7days old^49^^,^^50^ and higher luminal concentrations of fosfomycin.

The CSF data available from this study were not sufficiently rich to support estimation of Q2. Instead, Q2 was fixed using adult prior information. Kuhnen *et al*.^16^ report full plasma and CSF concentration–time profiles after both 5 g (*n *=* *35 subjects) and 10 g (*n *=* *5 subjects) IV fosfomycin. The mean age of the subjects in Kuhnen *et al*.^16^ was 47 years (range = 18 to 69) and each patient obtained an intra-operative or therapeutic CSF drain that was required for a neurosurgical indication. Both 5 and 10 g datasets were extracted from the publication and individually modelled; no covariates or between-subject variabilities were included in the modelling as only a single average plasma/CSF profile was published at each dose. The NONMEM code used to model the 5 g data can be found in the Supplementary data available at *JAC* Online and the associated fixed effects and residual errors, along with GOF plots, are provided in Figure S1. The average Q2 estimated from modelling the adult fosfomycin PK data^16^ was 0.017 L/h/70 kg (to three decimal places).

The final population estimate of CSF UPTK in the NeoFosfo trial population was 0.32 (%RSE = 12.0, 95% CI = 0.27–0.41). Nau *et al*.^51^ previously reported that in adults with uninflamed or mildly inflamed meninges fosfomycin AUC_CSF_/AUC_S_ is 0.18 (0.09–0.27). Since progression from sepsis to meningitis in neonates can be rapid, and less overt than in older children, fosfomycin’s good CSF penetration in this population is supportive of its potential role in empirical regimens for neonatal sepsis.

Whilst we use demographic data from the neonatal observational study to simulate pre-term neonates and those weighing <1500 g (see Figures S12 and S13) that were not included in our model building population, the mechanistic covariates used with biological priors on allometric weight and PMA scaling give some confidence in this extrapolation. We are assuming, however, that our observed PNA maturation effect will follow a similar trajectory in smaller neonates and, whilst this assumption may be reasonable, it could only be fully confirmed by collecting further data in this population. Nevertheless, this analysis indicates that reducing the dose in the first week of life due to short-term PNA maturation is required regardless of GA and/or weight.

A caveat specific to the oral TA predictions concerns the use of a constant Ka in all subjects. Following oral dosing the Ka in an individual will have a significant impact on the shape of the oral PK profile and therefore this does introduce an element of additional uncertainty compared with the IV TA predictions.

Finally, further *in vitro* work to better define the target AUC/MIC ratio required to achieve a 2 log kill against pathogens typically responsible for neonatal sepsis is needed, alongside work to define relevant MIC breakpoints for bacteraemia and meningitis. Confirmatory PK data in pre-term neonates would also be useful to make firmer conclusions on dosing in this population.

### Conclusions

To the best of our knowledge, this is the first study to report model-based oral bioavailability from cross-over data in a neonatal antibiotic study and the first report of neonatal fosfomycin CSF penetration. PNA in addition to PMA was needed to describe immediate postnatal changes in CL distinct from gestational effects. We also establish a positive relationship between PR_i_ and CSF uptake of fosfomycin. The planning of follow-up fosfomycin trials in neonates will benefit from our model and TA simulations, which can be used to inform selection of a neonatal IV dose based on an infant’s PMA, PNA and weight, and, where relevant, an oral step-down dose personalized using likely pathogen MIC at 48 h.

Our prediction of the PK in smaller pre-term neonates (<1500 g, <34 weeks GA) is based on extrapolation of fixed covariate effects that are well established for renally cleared drugs;^52^ however, it is important that these predictions are confirmed in a follow-up prospective trial.

Further *in vitro* work to better define target AUC/MIC ratios to achieve a 2 fold log kill against pathogens typically responsible for neonatal sepsis, alongside work to define relevant MIC breakpoints for bacteraemia and meningitis, would be beneficial.

## Supplementary Material

dkab083_Supplementary_Data
