# Supplementary material for: IV and oral fosfomycin pharmacokinetics in neonates with suspected clinical sepsis
Source: J Antimicrob Chemother. 2021 Apr 14;76(7):1855–64. doi: 10.1093/jac/dkab083 (PMC8212774; doi:10.1093/jac/dkab083)
Supplement: dkab083_Supplementary_Data [file dkab083_supplementary_data.docx]

SUPPLEMENTARY DATA

## Adult CSF Modelling:


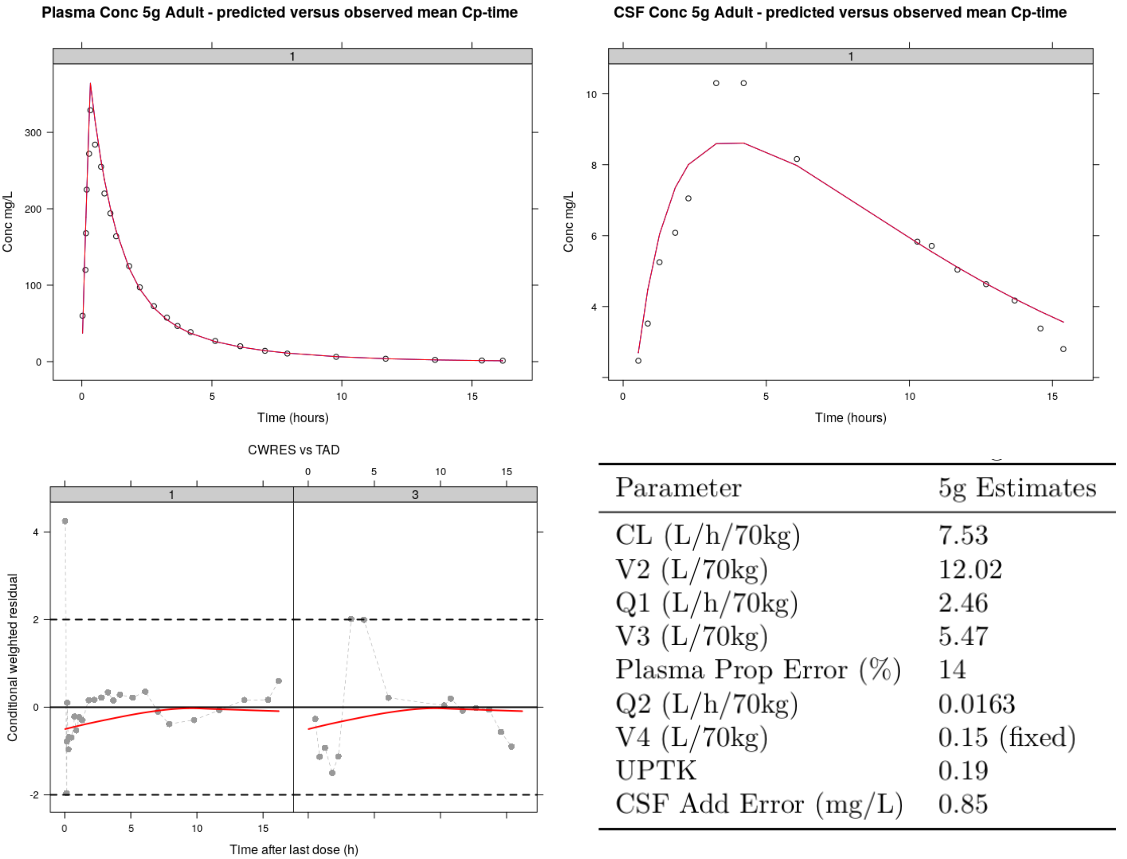


Figure S1: Adult CSF Modelling. Data extracted from Kuhnen et al, Penetration of fosfomycin into cerebrospinal fluid across non-inflamed and inflamed meninges, Infection 15, 422–424 (1987).

## Subject 1 to 30 observed plasma and CSF concentration time data:


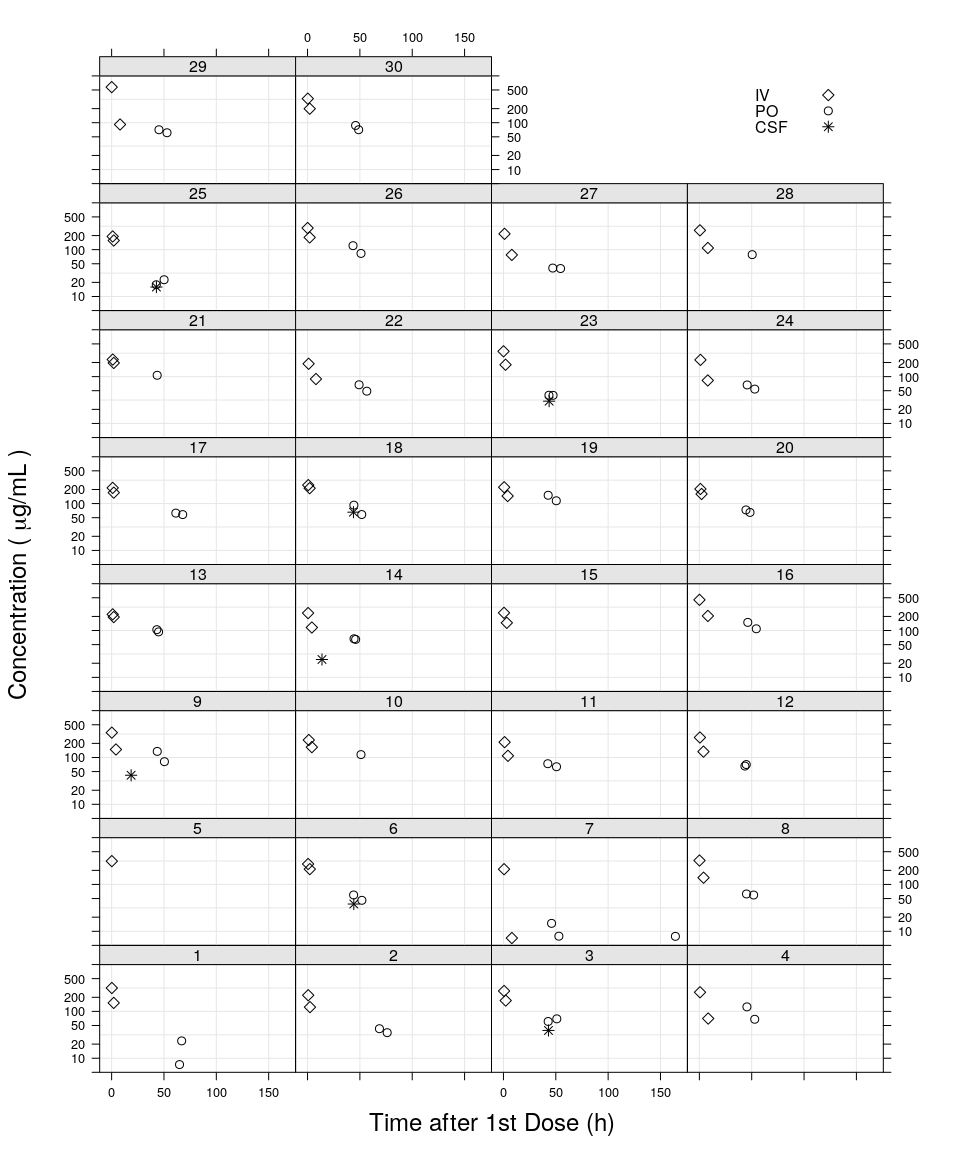


Figure S2: Fosfomycin PK population - Subjects 1 to 30. Open diamonds; IV plasma levels, open circles; PO plasma levels, asterix; CSF levels.

## Subject 31 to 60 observed plasma and CSF concentration time data:


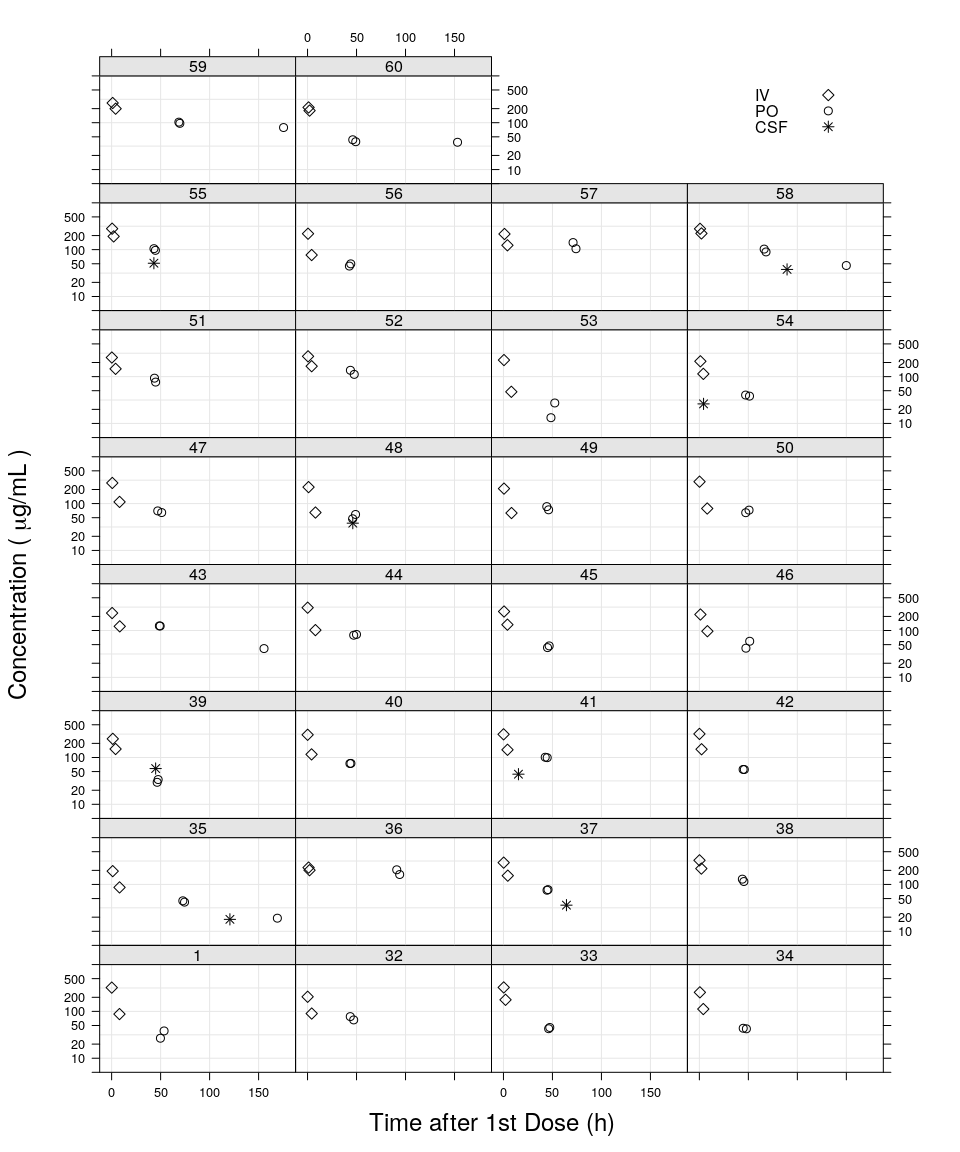


Figure S3: Fosfomycin PK population - Subjects 31 to 60. Open diamonds; IV plasma levels, open circles; PO plasma levels, asterix; CSF levels.

## Covariate correlations:


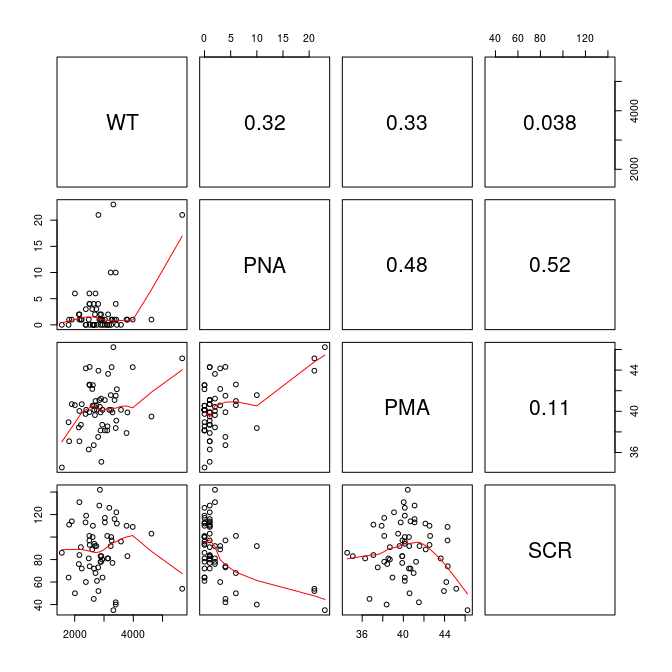


Figure S4: Baseline covariate correlations, plots in lower triangle, correlation coefficients in upper triangle

## Final Structural PK Model:


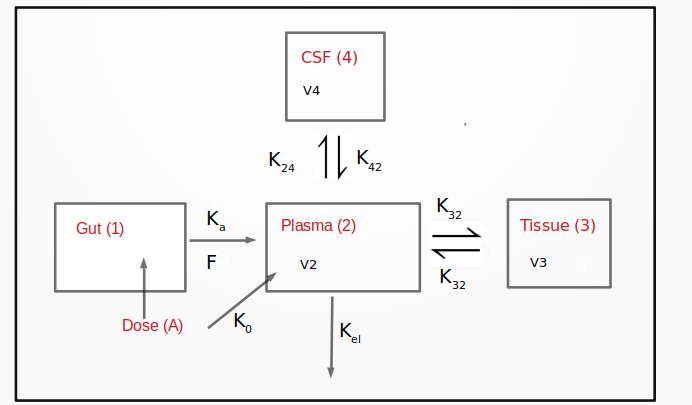


Figure S5: Structural PK Model including CSF Compartment. Intercompartmental clearances and volumes are derived from the following rate constants as follows; K20=CL/V2, K23=Q1/V2, K32=Q1/V3, K24=Q2*UPTK/V2, K42=Q2/V4, K12=Ka and K0=Intravenous dose rate (nominally 6000mg/kg/h)

## Goodness of Fit Plots:


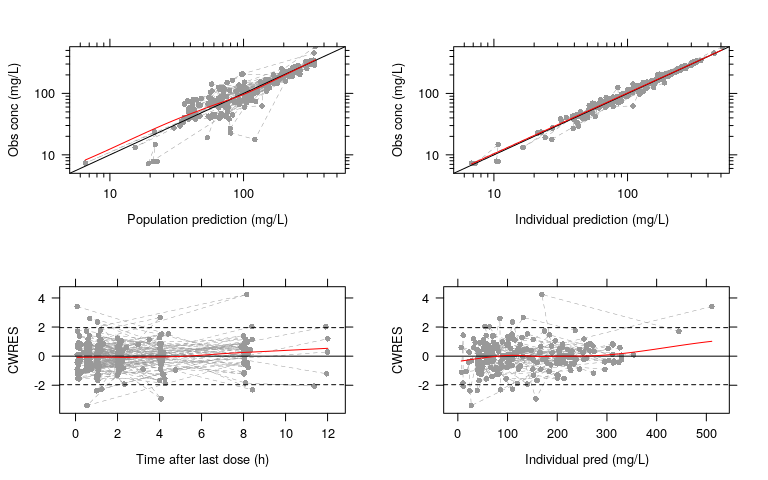


Figure S6: Plasma Godness of Fit Plots


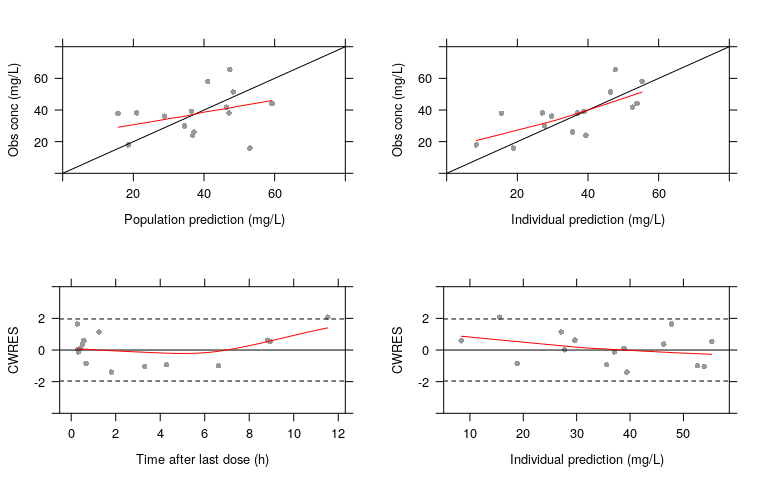


Figure S7: CSF Godness of Fit Plots

## Individual Plots - Subject 1 to 30 observed and predicted plasma and CSF concentration time data:


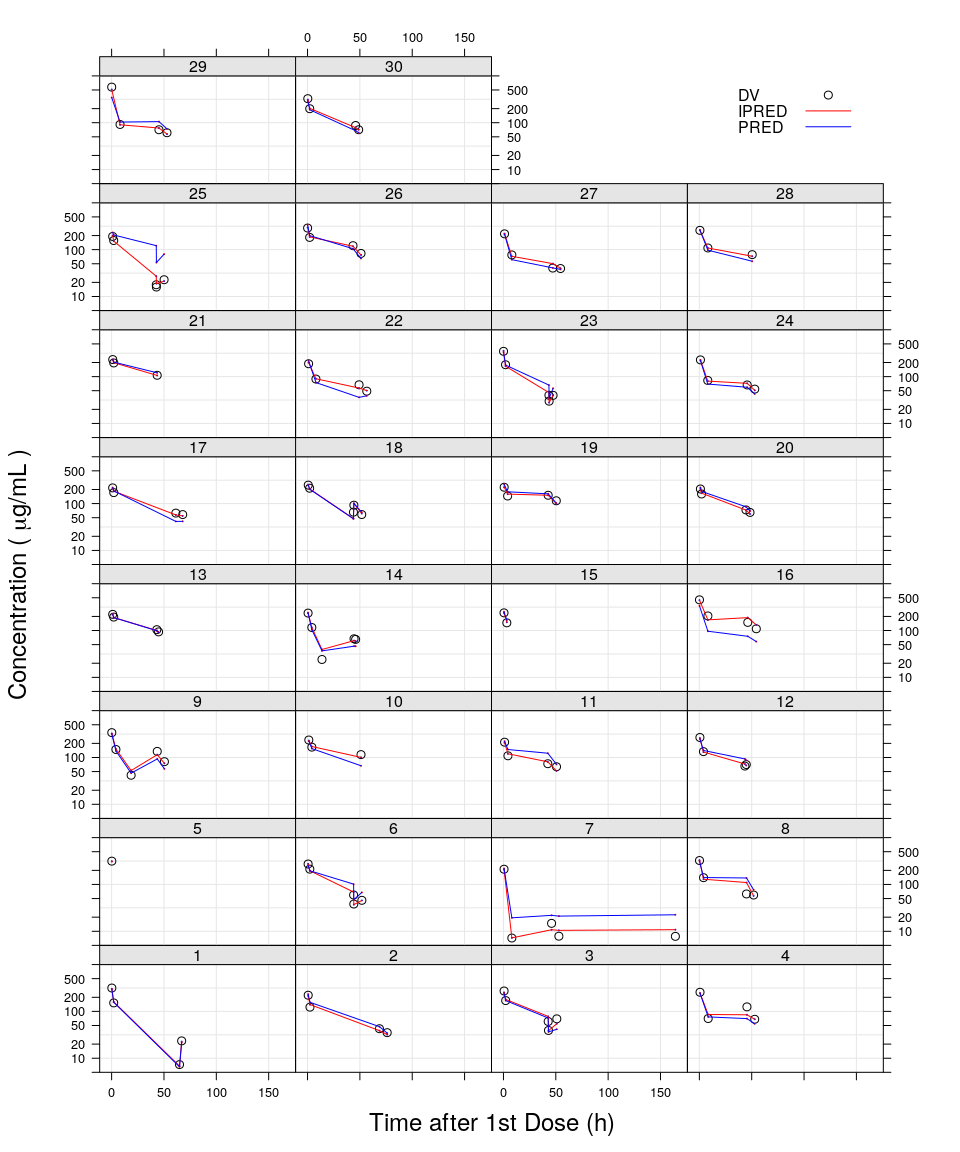


Figure S8: iplots subject 1 to 30. Black circle = observed data, blue line = population prediction, red line = individual prediction.

## Individual Plots - Subject 31 to 60 observed and predicted plasma and CSF concentration time data:


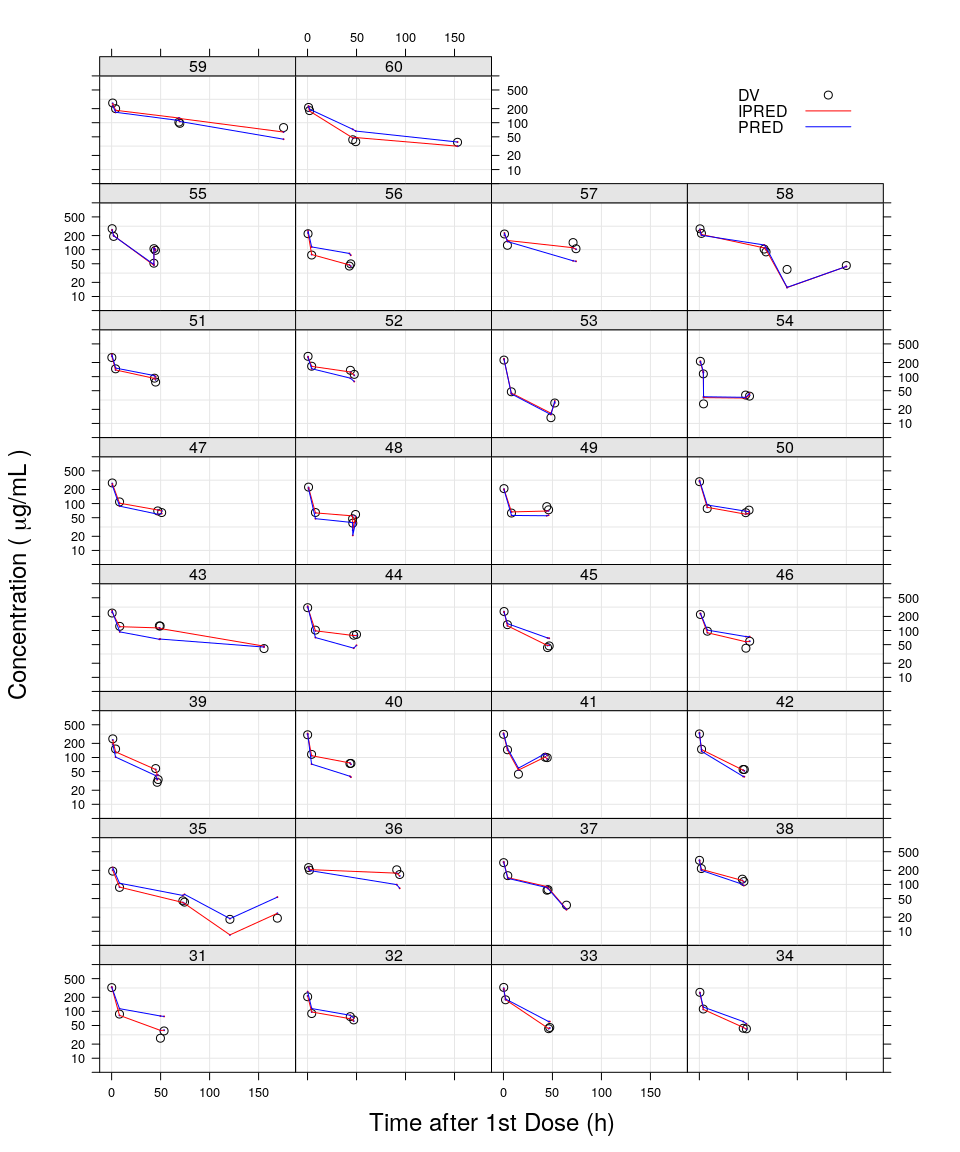


Figure S9: iplots subject 31 to 60. Black circle = observed data, blue line = population prediction, red line = individual prediction.

## NONMEM Code - NeoFosfo Model:

$PROBLEM Fosfomycin neonatal pharmacokinetics
$INPUT ID SEX GA AGE WT AMT TIME MATRIX DV PROTEIN MDV CMT ROUTE
 RATE TAD PNADAYS PNAYEARS TSCR PMAWEEKS SCR Na PNADAYS2
 PNASG PMA tCrea dCrea PNASGC flag
$DATA neofosfo.pk.200208.csv IGNORE=@
$SUBROUTINE ADVAN5 TRANS1
$MODEL COMP(1) ; oral absorption
 COMP(2) ; central compartment
 COMP(3) ; periperal compartment
 COMP(4) ; CSF compartment
$PK
;------ Covariate functions
 WTCL = (WT / 70000)**0.75 ; weight scaling for CL and Q terms
 WTV = (WT / 70000) ; weight scaling for volume terms
 MAT = PMA**3.4 / (47.7**3.4 + PMA**3.4) ; Rhodin 2008 GFR maturation model
 CLPNA = THETA(7) + (1 - THETA(7)) * (1 - EXP(-PNASGC * THETA(8))) ; CL post natal age change
;------ Typicl value of parameters
 TVCL = THETA(1) ; clearance
 TVV2 = THETA(2) ; central volume, allometrically scaled
 TVQ1 = THETA(3) ; Inter compartmental clearance C2 to C3
 TVV3 = THETA(4) ; Peripheral volume
 TVKA = THETA(5) ; Absorption rate constant
 TVF1 = THETA(6) ; Oral Bioavailiability
 TVQ2 = THETA(9) ; Intercomparmental clearance between C2 and C4
 TVUP = THETA(10) ; Fraction uptake into CSF compartment
;------ MU modelling
 MU_1 = DLOG(TVCL)
 MU_2 = DLOG(TVV2)
;------ Individual parmaeters
 CL = DEXP(MU_1 + ETA(1)) * WTCL * MAT * CLPNA
 V2 = DEXP(MU_2 + ETA(2)) * WTV
 Q1 = TVQ1 * WTCL
 Q2 = TVQ2 * WTCL ; no eta on Q2 as not enough data
 V3 = TVV3 * WTV
 V4 = 0.15 * WTV ; fixed volume of CSF in 70Kg adult to estimate uptake fraction
 KA = TVKA
;----- Oral bioavailability logit
 BIO1 = DLOG(TVF1 / (1 - TVF1)) ; (-inf,inf)
 BIO2 = BIO1 + ETA(3) ; iiv now on normal scake
 F1 = 1 / (1 + DEXP(-BIO2)) ; back to (0, 1)
;------ CSF uptake fraction logit
 UPTK1 = DLOG(TVUP/(1 - TVUP)) ; (-inf,inf)
 UPTK2 = UPTK1 * (1 + THETA(11) * (PROTEIN - 0.94)) ; Median protein = 0.94
 UPTK = 1 / (1 + DEXP(-UPTK2)) ; back to (0, 1)
;------ Rate constants
 K20 = CL/V2
 K23 = Q1/V2
 K32 = Q1/V3
 K24 = Q2*UPTK/V2
 K42 = Q2/V4
 K12 = KA
 S2 = V2
 S4 = V4

$ERROR
CP = A(2) / V2
CCSF = A(4) / V4
;------ Plasma predictions
IF(CMT==2) IPRED = CP
IF(ROUTE==1) Y = IPRED * (1 + EPS(1)) ; EPS(1) is proportional residual error on IV
IF(ROUTE==2) Y = IPRED * (1 + EPS(2)) ; EPS(2) is proportional residual error on PO
;------ CSF predictions
IF(CMT==4) IPRED = CCSF
IF(CMT==4) Y = IPRED + EPS(3) ; residual error term for csf, additive error model
;------ Calculate IWRES
 IRES = DV-IPRED
 IF(ROUTE==1) SD = SQRT(SIGMA(1,1)) * IPRED ; IV
 IF(ROUTE==2) SD = SQRT(SIGMA(2,2)) * IPRED ; PO
 IF(CMT==4) SD = SQRT(SIGMA(3,3)) ; CSF
 IF(SD==0) SD = 1
 IWRES = IRES/SD

;------ Final parameter estimates
 $THETA 8.94306 ; 1. TVCL
 $THETA 19.1087 ; 2. TVV2
 $THETA 8.00673 ; 3. TVQ1
 $THETA 7.53073 ; 4. TVV3
 $THETA 0.0987403 ; 5. TVKA
 $THETA 0.478167 ; 6. TVF1
 $THETA 0.449443 ; 7. M - fraction of clearance on day of birth
 $THETA 0.116786 ; 8. N - rate of maturation post birth
 $THETA 0.017 FIX ; 9. Intercomparmental clearance Q2 between C2 and C4
 $THETA 0.320868 ; 10. uptake into CSF,fractional
 $THETA -0.952217 ; 11. protein covariate
;------ Interindividual parameter variability
 $OMEGA 0.0599312 ; variance for ETA(1), initial estimate
 $OMEGA 0.0202154 ; variance for ETA(2), initial estimate
 $OMEGA 0.269266 ; variance for ETA(3), initial estimate
;------ Residual error
 $SIGMA 0.00592027 ; variance prop res error, IV plasma, initial estimate
 $SIGMA 0.0344364 ; variance prop res error, PO plasma, initial estimate
 $SIGMA 117.851 ; variance add res error, CSF, initial estimate

$ESTIMATION METHOD=1 INTER MAXEVAL=9999 PRINT=1 NOABORT
;------ standard error of estimate is calculated
$COVARIANCE
;------ Table output
$TABLE ID TIME CMT TAD ROUTE MATRIX MDV IPRED IWRES CWRES NPDE
 ESAMPLE=300 NOPRINT ONEHEADER FILE=sdtab355
$TABLE ID CL V2 V3 V4 Q1 Q2 KA F1 ETAS(1:3) NOPRINT NOAPPEND
 ONEHEADER FILE=patab355
$TABLE ID GA AGE WT PROTEIN PNADAYS PNAYEARS TSCR PMAWEEKS SCR
 NOPRINT NOAPPEND ONEHEADER FILE=cotab355
$TABLE ID SEX ROUTE NOPRINT NOAPPEND ONEHEADER FILE=catab355

## Simulation Population Demographics:


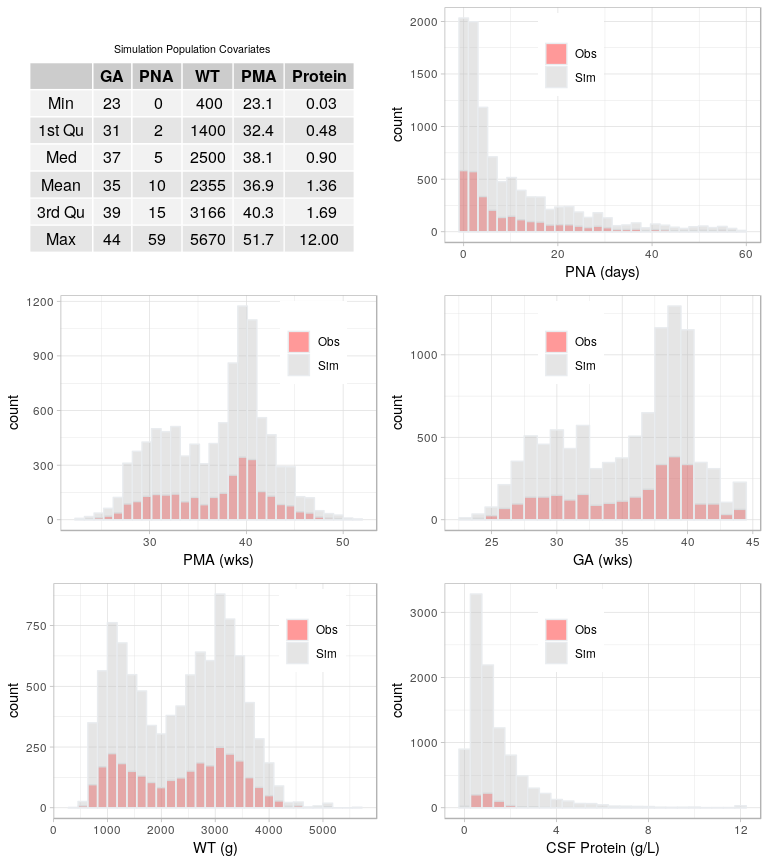


Figure S10: Hypothetical Simulation Population Demographics (n=10,000)

## Simulation Summary PK Table:


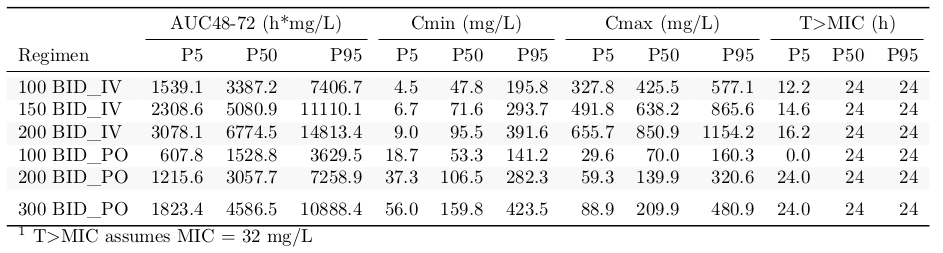


Table S1: Simulated steady state PK summary table: Full simulation population

## Plasma fT>MIC Target Attainment Plots - Full Population:


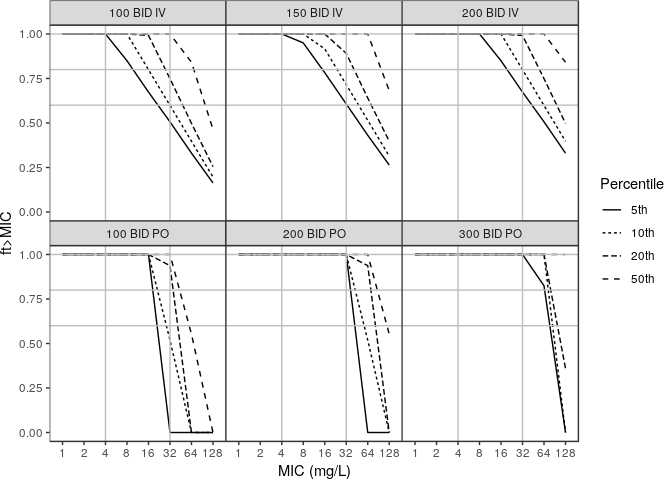


Figure S11: Target Attainment, Plasma fT>MIC, IV and PO Simulations, Full Population

## NONMEM Code - Adult CSF Modelling:

$PROBLEM Adult Fosfomycin CSF Modelling (5 gram dose n = 35)
$INPUT TIME TAD DV AMT MDV CMT ROUTE ID WT PNADAYS2 PMAWEEKS RATE
$DATA adult.csf.5g.b.csv IGNORE=@
$SUBROUTINE ADVAN5 TRANS1
$MODEL
COMP(1) ; central compartment
COMP(2) ; periperal compartment
COMP(3) ; CSF compartment
$PK
;------ Typicl value of parameters
TVCL = THETA(1) ; clearance
TVV1 = THETA(2) ; central volume, allometrically scaled
TVQ = THETA(3) ; Inter compartmental clearance C1 to C2
TVV2 = THETA(4) ; Peripheral volume V2
TVQ1 = THETA(5) ; Intercomparmental clearance between C1 and C3
TVUP = THETA(6) ; Uptake into CSF compartment
;------ MU modelling
MU_1 = DLOG(TVCL)
MU_2 = DLOG(TVV1)
MU_3 = DLOG(TVQ)
MU_4 = DLOG(TVV2)
;------ Individual parmaeters
CL = DEXP(MU_1+ETA(1))
V1 = DEXP(MU_2+ETA(2))
Q = DEXP(MU_3+ETA(3))
Q1 = TVQ1 ; no eta on Q2 as not enough data
V2 = DEXP(MU_4+ETA(4))
V3 = 0.15 ; fixed volume of 150 mL CSF in 70Kg adult
; CSF uptake
UPTK1 = DLOG(TVUP/(1 - TVUP)) ; logit transformation (-inf,inf)
UPTK2 = UPTK1 ; FOR COVARIATE MODELLING - not needed here
UPTK = 1 / (1 + EXP(-UPTK2)) ; back to (0, 1)
;------ Rate constants
K10 = CL/V1
K12 = Q/V1
K21 = Q/V2
K13 = Q1*UPTK/V1
K31 = Q1/V3
S1 = V1
S3 = V3
$ERROR
CP = A(1) / V1 ; plasma
CCSF = A(3) / V3 ; CSF
;------ Statistical model
IPRED = 0
IF(CMT==1)IPRED=A(1)/V1
IF(CMT==3)IPRED=A(3)/V3
IF(CMT==1) Y = IPRED * (1 + EPS(1)) ; proportional residual error model for plasma
IF(CMT==3) Y = IPRED + EPS(2) ; additive residual error model for CSF
;------ Calculate IWRES
IRES = DV-IPRED
IF(CMT==1) SD = SQRT(SIGMA(1,1))*IPRED
IF(CMT==3) SD = SQRT(SIGMA(2,2))
IF(SD==0) SD=1
IWRES = IRES/SD

;------ Final parameter estimates
$THETA 7.53458 ; 1. TVCL
$THETA 12.0166 ; 2. TVV2
$THETA 2.45879 ; 3. TVQ1
$THETA 5.47297 ; 4. TVV3
$THETA 0.0163026 ; 5. Intercomparmental clearance Q2 between C2 and C4
$THETA 0.194977 ; 6. fractional uptake into CSF
;------ Interindividual parameter variability
$OMEGA 0 FIX ; variance for ETA(1), initial estimate
$OMEGA 0 FIX ; variance for ETA(2), initial estimate
$OMEGA 0 FIX ; variance for ETA(3), initial estimate
$OMEGA 0 FIX ; variance for ETA(4), initial estimate
;------ Residual error
$SIGMA 0.020363 ; variance prop res error, plasma, initial estimate
$SIGMA 0.717904 ; variance add res error, csf, initial estimate
;
$ESTIMATION METHOD=1 INTER MAXEVAL=9999 PRINT=1 NOABORT; calculation method
;
$COVARIANCE
;
$TABLE ID TIME TAD ROUTE MDV IPRED IWRES CWRES NPDE ESAMPLE=300 NOPRINT
 ONEHEADER FILE=sdtab41
$TABLE ID CL V1 V2 Q Q1 UPTK ETAS(1:4) NOPRINT NOAPPEND ONEHEADER
 FILE=patab41
$TABLE ID WT NOPRINT NOAPPEND ONEHEADER FILE=cotab41
$TABLE ID ROUTE NOPRINT NOAPPEND ONEHEADER FILE=catab41

## Plasma AUC:MIC Target Attainment Plots - Sub Populations IV:


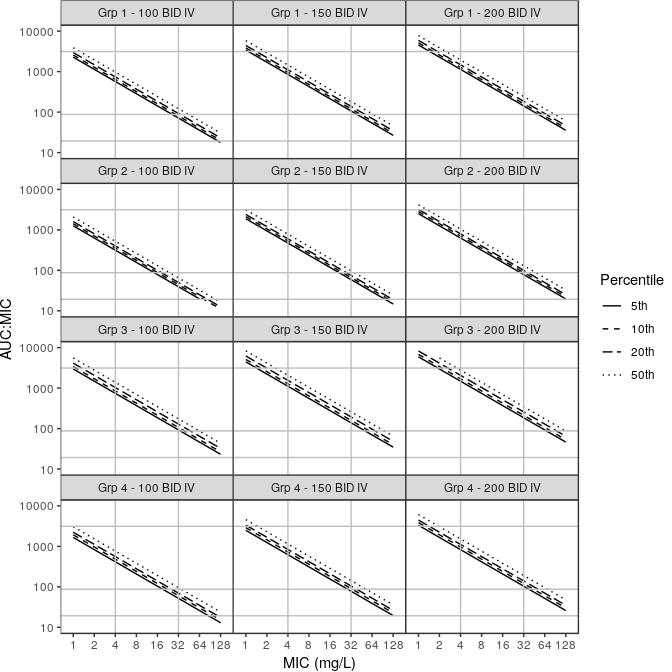


Figure S12: Target Attainment, Plasma AUC:MIC, IV dosing, Neonatal Sub-Populations. Group 1: WT>1.5kg+PNA<=7days (n=4391), Group 2: WT>1.5kg+PNA>7days (n=2798), Group 3: WT<=1.5kg+PNA<=7days (n=1534), Group 4: WT<=1.5kg+PNA>7days (n=1277). Groups 1 and 2 represent patients similar to those fitting our inclusion criteria. Groups 3 and 4 represent an extrapolation to pre-term neonates that were not studied in our population.

## Plasma AUC:MIC Target Attainment Plots - Sub Populations PO:


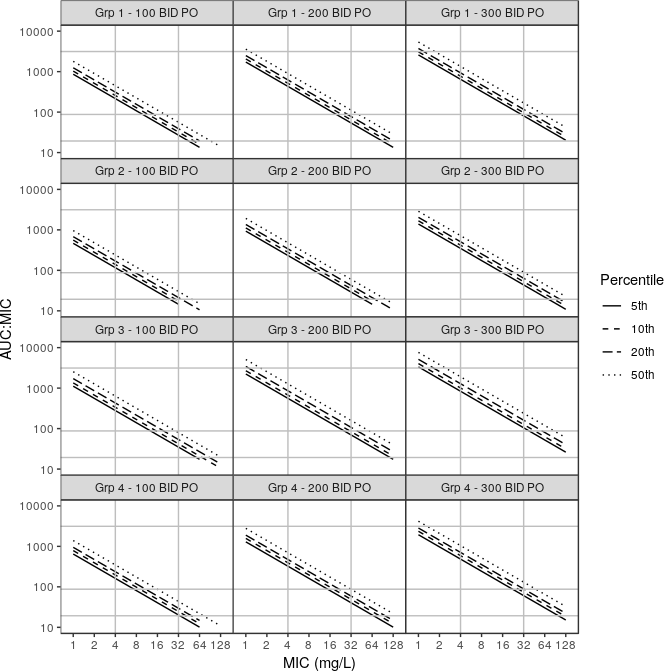


Figure S13: Target Attainment, Plasma AUC:MIC, PO dosing, Neonatal Sub-Populations. Group 1: WT>1.5kg+PNA<=7days (n=4391), Group 2: WT>1.5kg+PNA>7days (n=2798), Group 3: WT<=1.5kg+PNA<=7days (n=1534), Group 4: WT<=1.5kg+PNA>7days (n=1277). Groups 1 and 2 represent patients similar to those fitting our inclusion criteria. Groups 3 and 4 represent an extrapolation to pre-term neonates that were not studied in our population.

## Supplementary Analytical Information:

Analytical method: Protein precipitation and liquid chromatography with tandem mass spectrometry detection (LC-MS/MS)

Assay procedure: 20 µL plasma calibrator / quality control sample and 150 µL internal standard solution were added into 1.5 mL polypropylene tubes. The tubes were mixed for a minimum of 10 minutes, centrifuged for 2 minutes at 13,000 rpm and the supernatant was transferred to 96-well plate and 400 µL of de-ionised water was addedd. All the wells were mixed using 2-3 aspitating-dispensing cycles with an 8-channel pipette. 96-well plastes were submitted for analysis by LC-MS/MS.

Chromatographic conditions: Analysis by ultra-high-performance liquid chromatography using Hypercarb 30 x 2.1 mm, 3 µm column held at 50 °C. The mobile phase consisted of 0.1 % formic acid in methanol and 0.1 % formic acid in de-ionised water at a flow rate of 0.6 mL/min. Injection volume was 10 µL. Detection was by tandem mass spectromety on a TQD in positive mode: 138.9124 → 56.9575 for fosfomycin and 142.0319 → 78.8702 for fosfomycin-^13^C_3_. Additionaly qualifier ions 138.9124 → 120.9963 for fosfomycin in positive mode and 139.9042 for fosfomycin-^13^C_3_ in negative mode were detected.

Rention times: Fosfomycin and fosfomycin-^13^C_3_ eluted in less than 9.1 minutes.

Stability: Fosfomycin short term stability at room temperature stored for 24 and 48 h indicated 95.097 to 107.731 % of accuracy for low and high concentrations. Fosfomycin short term stability at +4 °C indicated accuracy of 98.794 to 111.182 % over the period of 24 to 48 h. The accuracy of fosfomycin in three freeze/thaw cycles ranged frim 95.068 to 102.571 %. The accuracy of fosfomycin long-term stability plasma samples ranged from 90.623 to 100.913 % after the storage period of 4 and 6 months. All measured quality control samples remained well within the ranges allowed.

Conclusion: This method showed satisfactory analytical performances for the determination of fosfomycin in human plasma over the calibration range of 5 microg/mL to 2000 microg/mL.
